# Effectiveness of an anti-inflammatory diet intervention and cognitive behavioural therapy in endometriosis: protocol for a randomised controlled clinical trial

**DOI:** 10.1136/bmjopen-2026-116964

**Published:** 2026-05-26

**Authors:** Emma Huijs, Loutje van der Sman, Lieke Wirken, Heidi SMJ Delcliseur, Esther G Winter, Nicole M de Roos, Renate G van der Molen, Joukje M Oosterman, Paola Viganò, Andrew W Horne, Marie-Madeleine Dolmans, Janneke S Hoogstad-van Evert, Annemiek W Nap, EUmetriosis Working Group, Martina Abodi

**Affiliations:** 1 Department of Obstetrics & Gynaecology, Radboud University Medical Center, Nijmegen, GE, the Netherlands; 2 Department of Laboratory Medicine, Laboratory for Medical Immunology, Radboud University Medical Center, Nijmegen, the Netherlands; 3 Department of Medical Psychology, Amphia Hospital, Breda, the Netherlands; 4 Department of Medical Psychology, Rijnstate Hospital, Arnhem, the Netherlands; 5 Division of Human Nutrition and Health, Wageningen University & Research, Wageningen, the Netherlands; 6 Donders Institute for Brain Cognition and Behaviour, Radboud University, Nijmegen, the Netherlands; 7 Department of Obstetrics & Gynaecology, Fondazione IRCCS Ca’ Granda Ospedale Maggiore Policlinico, Milan, Italy; 8 Institute of Regeneration and Repair, The University of Edinburgh MRC Centre for Reproductive Health, Edinburgh, UK; 9 Gynaecology Research Laboratory, Department of Gynaecology, Institut de Recherche Expérimentale et Clinique, Université catholique de Louvain, Louvain-la-Neuve, Belgium; 10 Gynaecology Department, Cliniques universitaires Saint-Luc, Brussels, Belgium; 11 Department of Obstetrics & Gynecology, Amphia Hospital, Breda, the Netherlands

**Keywords:** GYNAECOLOGY, NUTRITION & DIETETICS, IMMUNOLOGY, Quality of Life, Inflammation, PAIN MANAGEMENT

## Abstract

**ABSTRACT:**

**Introduction:**

Treatment for women with endometriosis is only partially or temporarily effective. Moreover, medical hormonal treatment is associated with debilitating side effects and interferes with fertility, while surgery has a relatively high risk of complications. Meanwhile, women with endometriosis show increasing interest in implementing lifestyle interventions to alleviate symptoms and improve health-related quality of life (HRQoL). Integrating these lifestyle interventions can provide a holistic approach to the treatment of this debilitating disease. However, scientific evidence supporting the effectiveness of these interventions is limited. This study is designed to investigate the effectiveness of two lifestyle interventions and the combination of both: an anti-inflammatory diet intervention (AIDI) could improve immune cell function and reduce inflammation, resulting in improved HRQoL and alleviating pain. In addition, the integration of cognitive behavioural therapy (CBT) aims to provide insight into pain mechanisms and coping with pain, and to assist in sustaining dietary adjustments.

**Methods and analysis:**

The Pain in Endometriosis And the Relation to Lifestyle (PEARL) study is a five-arm randomised controlled trial with a pre-post factorial design with two factors: an AIDI and CBT. The study population will consist of 250 premenopausal women, of whom 200 are diagnosed with endometriosis and experience pain symptoms and 50 are healthy controls (HC). Women with endometriosis will be recruited from one academic tertiary and five secondary hospitals in the Netherlands. They will be randomised (1:1:1:1) among four intervention groups: standard care (SC) (SC group), SC and an AIDI (SC + AIDI group), SC and CBT (SC + CBT group), and SC, AIDI and CBT (SC + AIDI + CBT group). Women with endometriosis will visit the hospital twice during the intervention period, at the start (T0) and end (T2) of the 13-week intervention period. HC will not undergo any of the interventions and will have one hospital visit (T0). Participants will complete questionnaires regarding pain symptoms, HRQoL, physical activity level, sleep, diet quality, pain cognitions, and stress at T0 and T2. Furthermore, they are instructed to collect menstrual effluent, a vaginal swab and a faecal sample. During the study visits, peripheral blood will be drawn and scalp hair samples will be taken. The primary outcome is average pain, measured using a numerical rating scale. Secondary outcomes focus on HRQoL, inflammation, immune system characteristics, vaginal- and gut microbiome, and hair cortisol levels. These are considered to reflect potentially underlying mechanisms of the effect of both interventions on the primary outcome. Biological samples and questionnaires of women with endometriosis and HC will be compared to establish the differences in secondary outcomes.

**Ethics and dissemination:**

This study protocol has been approved (approval number: NL86247.091.24) by the METC Oost-Nederland from Radboud University Medical Centre on July 11, 2024. Prior to participation, participants are required to provide informed consent. The results will be widely disseminated through scientific peer-reviewed journals, and presentation to a broad audience in scientific meetings, congresses, patient meetings, as well as in policy-relevant forums.

**Trial registration number:**

NCT06332560.

**STRENGTHS AND LIMITATIONS OF THIS STUDY**

- The combination of two lifestyle interventions, an anti-inflammatory diet intervention (AIDI) and cognitive behavioural therapy (CBT), is an innovative approach.
- The AIDI and CBT protocol were carefully designed by a team of dietitians and psychologists, and will be personalised, which will improve the adherence to the intervention as well as its effect.
- The integrative approach of objective and subjective data will offer insights into both biological and psychological mechanisms affecting endometriosis.
- Due to the nature of the interventions, participants cannot be blinded to group allocation.
- As the AIDI and CBT are personalised, the intervention is not completely standardised.

## Introduction

 Many individuals diagnosed with endometriosis continue to experience recurrent symptoms even after undergoing surgery or using hormonal suppression.^1^ Despite being the most prevalent benign gynaecological disorder, affecting approximately 10% of reproductive age individuals (from here referred to as reproductive age women), endometriosis cannot be cured to this day.^1 2^ Therefore, self-management strategies such as lifestyle interventions become vital for long-term alleviating symptoms and improving health-related quality of life (HRQoL). These strategies can empower women with endometriosis to actively manage their symptoms, fostering greater autonomy and resilience.^3^ Ultimately, integrating these lifestyle interventions in the treatment of endometriosis provides a holistic approach in the managment of this debilitating disease.

Endometriosis is a chronic, oestrogen-dependent inflammatory disease characterised by the presence of endometrial-like tissue outside the uterine cavity.^1^ Mounting evidence implicates immune dysregulation and persistent inflammation in the pathophysiology and severity of symptoms.^4–7^ Endometriotic lesions recruit a large number and diversity of immune cells which contributes to increased production of cytokines, chemokines and growth factors. This results in an enhanced inflammatory environment in the peritoneal cavity.^8^ Furthermore, elevated oestradiol levels in endometriotic lesions further exacerbate the inflammatory environment.^9^ This inflammatory reaction stimulates the activity of nerve fibres in and around the endometriotic lesions, which eventually leads to peripheral sensitisation. In the long term, this may result in changes in the central nervous system leading to heightened pain perception, known as central sensitisation, potentially contributing to chronic pelvic pain,^10^ the hallmark symptom of endometriosis.^11^ Furthermore, endometriosis can present with dysmenorrhoea, dyspareunia, dysuria and dyschezia, as well as gastrointestinal symptoms, infertility, migraine, and fatigue.^12 13^ Besides the physical symptoms associated with endometriosis, this condition can also affect psychological well-being and impair economic productivity and social relationships, which negatively affects HRQoL.^14–16^

To alleviate pain symptoms and improve HRQoL, many women with endometriosis turn to self-management strategies. While dietary adjustments have been identified as one of the most used self-management strategies in reducing pelvic pain and gastrointestinal symptoms by women with endometriosis,^3^ there is no specific diet that consistently proves to alleviate symptoms more effectively than other dietary interventions.^17^ Furthermore, the scientific evidence regarding the effectiveness of dietary interventions remains of low quality, with a lack of large-scale intervention studies investigating the effect of these dietary interventions on endometriosis-related pain symptoms and HRQoL.^18^ In addition, it is unclear whether the experienced symptom reduction is attributable due to the self-empowerment effect^19 20^ or because pain symptoms may be alleviated due to a biological mechanism of nutrition on the body.

Several macro- and micronutrients have been shown to influence the immune status of an individual.^21 22^ A person’s inflammatory status can switch from pro- to anti-inflammatory. The Western diet, which is typically rich in processed foods, refined sugars, saturated fats, trans fats, and low in dietary fibre, is associated with immune dysregulation and low-grade chronic inflammation. In contrast, a diet rich in anti-inflammatory nutrients such as antioxidants, dietary fibre and omega-3 fatty acids from vegetables, fruits, whole grains, legumes, nuts, fatty fish, and olive oil is associated with beneficial effects on the immune system and reduced inflammatory responses.^23 24^ Therefore, an anti-inflammatory diet intervention (AIDI) may be effective in influencing the inflammatory status of women with endometriosis, resulting in the suppression of pain symptoms.

Similar to the biological and physical determinants of pain, psychological factors also contribute significantly to its experience,^25^ they play a crucial role in pain perception, often even surpassing the impact of physical symptoms. Women with endometriosis are prone to exhibit negative pain cognitions, including catastrophising and hypervigilance, which independently influence HRQoL.^26^ Furthermore, endometriosis is associated with increased stress levels. The relationship between stress and endometriosis is bidirectional, as, next to endometriosis leading to elevated stress levels, stress itself can exacerbate symptoms and disease progression.^27^ Stress and pain intensity are positively correlated; women with endometriosis reporting high stress levels exhibit significantly higher pain scores. Positive coping strategies are associated with better stress adaptation. Understanding the psychological aspects of pain can help women with endometriosis participate in their own pain management and assist clinicians in creating supportive environments for treatment.^28^ Cognitive behavioural therapy (CBT) is a psychological intervention which is proven effective in other chronic pain conditions and targets to help identify, modify and reduce negative cognitions and behaviours, which can help to decrease pain catastrophising, hypervigilance and anxiety.^29 30^

Thus, supporting women with endometriosis through lifestyle interventions may be crucial for symptom management and improving HRQoL. Therefore, the Pain in Endometriosis And the Relation to Lifestyle (PEARL) study examines the effects of two lifestyle interventions: an AIDI and CBT, on endometriosis-related symptoms and HRQoL. Both interventions are tested separately, as well as in conjunction with one another, to examine whether the combination of these interventions has an enhancing effect. To explore a possible underlying biological mechanism as a contributing factor for symptom improvement, the gut and vaginal microbiome, hair cortisol levels, inflammatory markers and immune cell composition in peripheral blood (PB), and menstrual effluent (ME) are being analysed before and after the interventions. In addition, this study aims to identify differences in these biomarkers between women with and without endometriosis to gain a deeper understanding of the pathogenesis of endometriosis. We assume that this will contribute to new insights in diagnostics and potential new treatment strategies for women with endometriosis.

## Methods and analysis

### Study design

**Figure 1:**
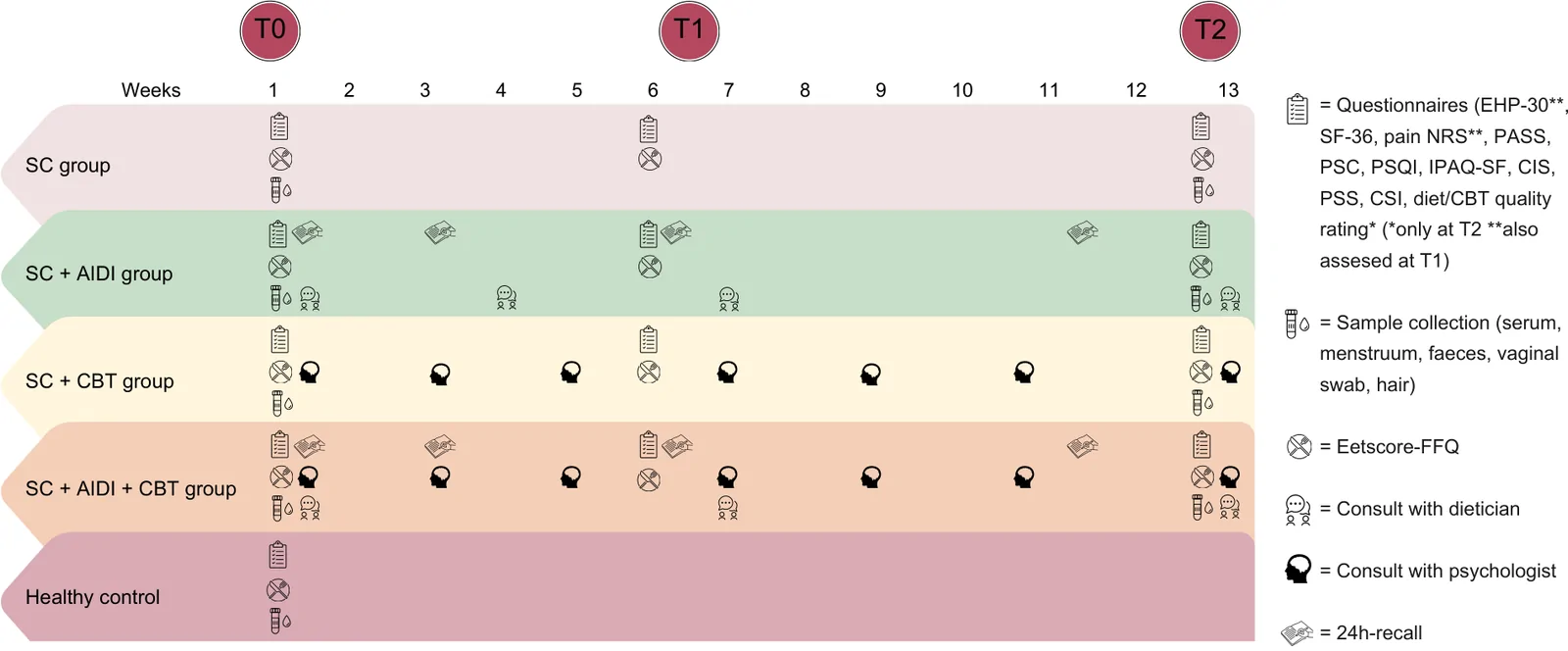
Overview of the study design. AIDI, anti-inflammatory diet intervention; CBT, cognitive behavioural therapy; CIS, Checklist Individual Strength; CSI, Central Sensitisation Inventory; EHP-30, Endometriosis Health Profile-30; FFQ, food frequency questionnaire; IPAQ-SF, International Physical Activity Questionnaire Short Form; NRS, Numerical Rating Scale; PASS, Pain Anxiety Symptom Scale; PSC, Pain Catastrophising Scale; PSQI, Pittsburgh Sleep Quality Index; PSS, Perceived Stress Scale; SC, standard care; SF-36, Short Form Healthy Survey-36.

This is a study protocol for a randomised controlled trial (RCT) with a pre-post factorial design with two factors: an AIDI and CBT. Women with and without endometriosis will be included, resulting in an RCT with five arms. Women with endometriosis will be randomised among four arms: a control group receiving standard care (SC group) and three intervention groups: receiving standard care and adhering to an AIDI (SC + AIDI group), receiving standard care + CBT (SC + CBT group), and receiving standard care, adhering to an AIDI, and receiving CBT (SC + AIDI + CBT group). The fifth arm is allocated to serve as the healthy control (HC) group (women without endometriosis diagnosis). HCs will not receive any of the interventions. An overview of the study design is presented in figure 1. This protocol was developed in accordance with the Standard Protocol Items: Recommendations for Interventional Trials reporting guidelines.^31^

### Patient and public involvement

Prior to the development of this study protocol, we conducted two qualitative studies in which the feasibility of an AIDI and CBT for women with endometriosis was identified.^32 33^ Information acquired in these studies was taken into account when designing both interventions used for this study protocol. In addition, members of endometriosis patient organisations from the Netherlands (‘the Dutch Endometriosis Society’), Croatia (‘I am 1 in 10’) and the UK (‘Endometriosis UK’) were involved in the development of this study protocol.

### Sample size calculation

The calculation of the required sample size for this RCT was conducted for the primary outcome, average pain intensity measured with a Numerical Rating Scale (NRS). Based on preliminary results of our single-arm explorative feasibility study and the RCT of Field *et al*, where a whole-food diet was implemented that restricts ultra-processed foods for pain management in chronic pain patients,^34^ we assume a pre-post correlation of r = 0.3. Although the single-arm trial found an effect (pre-post) of 1 SD, the expected effect size was set at 0.6 SD (–1.14).

For the CBT no pilot data were available. Therefore, the calculation of the effect size for this intervention was based on an RCT describing the efficacy of CBT in enhancing coping strategies, alleviating depression, stress, pain perception and improving the quality of life for women with endometriosis.^35^ In this study an effect of 0.55 SD was observed. For the current RCT this would translate to a similar effect of CBT as of the AIDI, so −1.14. We assume that the pre-post SD σ and correlation will be the same for all arms.

With 40 participants per arm, the precision for the main effects of AIDI and CBT yields an SD of 0.405, providing power greater than 80% for detecting an effect size of 1.14. However, the interaction effect has an SE of 0.57, ie, with a 95% CI for the estimated difference of ±1.12. This allows to observe an interaction effect in the order of magnitude of AIDI and CBT effects.

Based on our pilot-study we expect a dropout rate of 20–25%. Therefore, ten additional participants will be included in each arm of the RCT, resulting in a target sample size of 50 participants per arm, in total 200 women with endometriosis.

The evaluation of differences between HCs and women with endometriosis will be an explorative part of the study to generate preliminary insights. In the literature a sample size of 30–50 is described as a reasonable minimum sample size.^36^ Considering the potential drop-out and the group size in the other arms of the RCT, the total sample size for this RCT will be 250, of which 200 will be women diagnosed with endometriosis and 50 HC women.

### Study population and recruitment

Women with endometriosis will be recruited from six hospitals in the Netherlands, including Radboud University Medical Centre, Amphia Hospital, Rijnstate Hospital, Jeroen Bosch Hospital, Catharina Hospital and St. Antonius Hospital. In all hospitals, a multidisciplinary centre for the diagnosis and treatment of endometriosis is present. Patients diagnosed with endometriosis will be invited to participate in the RCT by their gynaecologist. Those who are interested will be referred to a researcher to assess eligibility based on inclusion and exclusion criteria. HC women will be recruited at the Radboud University, Radboud University Medical Centre and via social media channels.

Eligible participants are women of at least 17 years old, with a confirmed diagnosis of endometriosis or adenomyosis through ultrasound, MRI or surgery, and experiencing pain symptoms with an NRS score >4 (this inclusion criterion is not attributable for HC women). Furthermore, they are nulligravida, premenopausal, have a body mass index (BMI) between 18 and 30 kg/m², scalp hair longer than 4 cm, commit to not change their use of dietary supplements, and willing to follow an AIDI and CBT. Additionally, eligible participants need to be proficient in Dutch.

Exclusion criteria are smoking, following a vegan diet, use of immunosuppressive or psychotropic medication or recurrent miscarriages (>2), a diagnosis of Crohn’s disease, ulcerative colitis, short bowel syndrome, another chronic inflammatory disease, coeliac disease, an eating disorder or a serious mental illness (DSM-V diagnosis).

Before inclusion, a food frequency questionnaire (FFQ), the ‘Eetscore’-FFQ, and a demographic questionnaire will be administered to potential participants. Those who meet the inclusion criteria will be offered informed consent (online supplemental material 1) by an authorised researcher. An independent researcher can be contacted if participants wish to discuss the study with an expert who is not directly involved in the research project but has knowledge about all aspects of the study. Participants can withdraw consent at any time.

### Randomisation

After receiving signed informed consent, 1:1:1:1 computerised (Castor Electronic Data Capture (Castor EDC)) randomisation will take place to each of the four groups. An authorised researcher will be able to perform randomisation and have insight in the randomisation results. Stratification will be applied based on the following variables: hormonal use (yes/no), diagnosis of irritable bowel syndrome (yes/no), baseline Eetscore-FFQ outcome (<100/100 to 130), and baseline pain NRS average score (4 to 6/7 to 10). Randomisation within each stratum will be conducted to ensure comparability across groups for these factors.

This study is single blinded. Participants, nutritionist, and psychologists cannot be blinded due to the nature of the interventions, the assessor is blinded for the treatment allocation. Breaking the randomisation code will be done when patient’s health is at risk or when investigation is required by the sponsor, the Medical Ethics Committee (METC) Oost-Nederland or the monitor.

### Variables

The primary outcome of this study is the pain intensity score, measured with NRS, to assess the most intense pain and the average pain during last month, and endometriosis-specific pain symptoms (eg, dyspareunia, dyschezia, gastrointestinal symptoms) on a 0–10 point scale.

Secondary outcome variables are considered to reflect potentially underlying mechanisms of the effect of both interventions on the primary outcome. We will investigate the influence of the AIDI, CBT, and the combination of both interventions on inflammatory markers and immune cell composition in PB and ME, the vaginal and gut microbiome, and hair cortisol levels. Furthermore, HRQoL will be assessed with the Endometriosis Health Profile-30 (EHP-30) and Short Form Healthy Survey-36 (SF-36).

Demographic variables will be recorded including age, height, weight, waist circumference, BMI, marital status, education level, occupation, the method used to diagnose endometriosis, year of diagnosis, type of endometriosis, use of contraception, use of analgesics, use of medication, presence of subfertility, parity, diet history, history of eating disorders, history of (self-reported) intestinal disorders, history of DSM-V diagnosis, and if patients underwent psychological treatment prior to inclusion in this study.

Additional outcome variables are pain anxiety which will be measured with the Pain Anxiety Symptom Scale (PASS), and pain catastrophising which will be measured by the Pain Catastrophising Scale (PCS). Moreover, sleep and physical activity level are assessed to minimise confounding factors. Therefore, participants will fill in the Pittsburgh Sleep Quality Index to assess sleep quality and the International Physical Activity Questionnaire Short Form to assess physical activity level. Additionally, fatigue is measured using the Checklist Individual Strength. Self-reported stress levels will be measured with the Perceived Stress Scale. The central sensitisation of pain is assessed using the Central Sensitisation Inventory. To assess the consistency of participants in adhering to their diet and to determine whether participation in the study prompts premature dietary changes, the Eetscore-FFQ will be administered three times throughout the intervention period. Patients’ experience following the AIDI and/or CBT will be measured with an evaluation questionnaire.

All primary, secondary, and additional variables will be measured at baseline (T0) and after the intervention period of 13 weeks (T2). Women with endometriosis will visit the hospital twice during the intervention period, at the start (T0) and end (T2) of the study. HC will not undergo any of the interventions and will have one hospital visit (T0). During the study visits PB will be drawn, scalp hair samples will be collected, and participants will hand in ME, a vaginal swab, and faecal sample.

Halfway through the study period (T1), for an interim measurement, women with endometriosis will fill in the Eetscore-FFQ, as part of an adherence measurement. In addition, they will be asked to complete the pain NRS and EHP-30.

To evaluate the long-term feasibility, cost-effectiveness and impact of the interventions, women with endometriosis will be asked to complete the pain NRS, EHP-30, and Eetscore-FFQ at 6 months after completing the study (T3).

### Sample collection and processing

#### Peripheral blood

PB is collected at T0 and T2. For plasma isolation, one 10 mL ethylene diamine tetra acetic acid (EDTA) tube (BD Vacutainer), and one 10 mL sodium heparin tube (BD Vacutainer) will be used. For the collection of serum isolation, one 4 mL serum tube (BD Vacutainer) will be used. Lastly, for peripheral mononuclear cell (PBMC) isolation, blood will be collected in two 10 mL EDTA tubes.

Tubes for serum and plasma collection will be centrifuged for 10 min at 20 °C 2204 rpm within 4 hours after collection. The collected samples will be stored in 1.4 mL round bottom tubes (Micronic) at −80 °C until further analysis.

PBMC will be isolated using the StraightFrom Whole Blood PBMC Isolation Kit (human, Miltenyi Biotec) according to the manufacturer protocol. In short, erythrocytes will be sedimented and removed using Sedimentation Buffer II with 5 mM EDTA in combination with Red Blood Cell removal antibodies from Sedimentation Kit II (human, Miltenyi Biotec). After centrifugation for 3 min at 20 °C 495 rpm, the supernatant will be collected and washed with MACS separation buffer. Granulocyte and erythrocyte will be depleted via magnetic separation. This will be done using Depletion MicroBeads and an autoMACS Separator. PBMCs will be frozen in RecoveryTM cell freezing medium (Gibco, Life Technologies) at a concentration of 10–20 × 10^6^ cells/mL, and will be stored at −80 °C for 24 hours before being transferred to liquid nitrogen until further analysis.

#### Menstrual effluent

Participants will be instructed to collect ME at home using a menstrual cup (AllMatters OrganiCup) at T0 and T2. The collection of ME starts after the initial spotting (ie, very light bleeding), and will be done for up to 30 hours. For the first and second 12 hours (total of 24 hours), the cup will be emptied in a collection tube containing RPMI1640 medium (ThermoFisher Scientific, Waltham, Massachusetts, USA) supplemented with 10% human pooled serum (HPS) (Zen-Bio, Durham, North Carolina, USA), 1 mM pyruvate (ThermoFisher Scientific), 2 mM GlutaMAX (ThermoFisher Scientific), 100 U/mL penicillin/100 µg/mL streptomycin (ThermoFisher Scientific), and 0.3% sodium citrate (Merck, Darmstadt, Germany). These samples will be stored at room temperature for a maximum of 48 hours until processing. A small fraction of the collected ME in the first 12 hours is separated in an empty 15 mL tube and will be stored at 4 °C until processing. The last 6 hours of the collected ME will be emptied in a 50 mL tube with RPMI1640 medium and stored at 4 °C until processing

To isolate menstrual mononuclear cells (MBMC), ME samples from the first 24 hours will be pooled and centrifuged for 5 min at 20 °C 1483 rpm. The medium will be removed and the pellet will be filtered using a 70 µm cell strainer (PluriSelect Life Science, Leipzig, Germany) by creating a vacuum using a connector ring (PluriSelect). A fraction of the filtered ME will be stored in PAXgene Blood RNA Tube (BD Bioscience) for transcriptomic analysis. The remaining ME will be 1:1 diluted in PBS+2% HPS. MBMC will be isolated according to the PBMC isolation procedure described above.

The other two tubes will be centrifuged for 10 min at 20 °C 2204 rpm. Supernatants from both tubes will be collected in 1.4 mL round bottom tubes and stored at −80 °C until further analysis of metabolites and epigenetic profiling of the liquid phase of the ME. The pellet of the 50 mL tube will be resuspended in 20% glycerol (Thermo Scientific Chemicals) and stored at −80 °C for epigenetic profiling of the solid phase of the ME.

#### Vaginal swab and faeces

Participants will be instructed to use the eNat vaginal swab (Copan Diagnostics) during the luteal phase of their menstrual cycle. Faeces will be collected in a faeces tube (76×20 mm, Sarstedt), maximum 24 hours prior to the study visit. Both samples will be stored at 4 °C until study visit. After the study visit, faeces and vaginal swab will be stored at −80 °C until DNA isolation using the DNeasy Blood & Tissue Kit (Qiagen) and subsequent microbiome analysis.

### Interventions

#### Standard care

The two primary components of standard care for endometriosis include the use of analgesics, like paracetamol or non-steroidal anti-inflammatory drugs or morphine. Moreover, another component is the use of hormonal therapies, such as oral contraceptives, progestogens or gonadotrophin‑releasing hormone analogues or antagonists. Standard care is individualised based on the participants’ needs and physicians’ choice.

#### Anti-inflammatory diet intervention

During the intervention period of 13 weeks, participants will receive a total of four consultations with a dietitian. One at T0, immediately prior to the start of the intervention, and three during the intervention period (beginning, middle (T1), and end (T2)). A 24h-recall will be done by the dietitian at T0, and three times during the AIDI. The information received from the 24h-recall at T0 will be used to create a personalised nutrition plan based on the AIDI, which will be further discussed during the in-person consultation at T0. Participants will furthermore receive a tailored dietary advice, along with an information folder containing recipes, food substitutions and two-week meal plans.

When a subject has questions or experiences issues concerning the dietary advice, she is allowed to request an extra phone consultation with the dietitian.

The AIDI that is used in this RCT is based on the Dutch Healthy Diet Index 2015 (DHD2015)^37^ and shares features with the EAT-Lancet, Mediterranean and Healthy Eating Index dietary patterns. It promotes a high intake of vegetables, fruits, legumes, nuts, fish, whole grains and dairy, while limiting red and processed meat, sugary drinks, salt, added sugars, and saturated fats.^38^ Compared with DHD2015, the AIDI is stricter regarding (red) meat intake, making it predominantly plant-based. Dietary guidelines are summarised in online supplemental table 1.

#### Cognitive behavioural therapy

For the CBT, a protocol has been developed in concordance with several psychologists having affinity with the treatment of women with endometriosis. This protocol is shown in online supplemental table 1. CBT will be provided by a trained psychologist with experience in the treatment of women with endometriosis. During the intervention period there will be in total seven individual CBT sessions. Psychologists will follow the protocol, but the treatment can be personalised based on the results of the PCS and PASS questionnaire. The first CBT session will take 1 hour and will take place face to face in the hospital, subsequent sessions will take approximately 45 min and may be done online.

In the first session, management of expectations towards the CBT (and AIDI) will be addressed. In the six other sessions, attention will be paid to the biological link between endometriosis-related pain and stress, relaxation training, cognitive stress management, sexual relationships, and the management of anxiety, catastrophising, and hypervigilance. When a participant is allocated to the SC + AIDI + CBT group, every CBT session will also focus on how to be able to adhere to the AIDI. In addition to the sessions provided by a psychologist, an online CBT module is available containing general information about psychological mechanisms of chronic pain and exercises on coping with pain in the context of behaviour, cognitions, and emotions. Participants in the SC + CBT and SC + AIDI + CBT group can use this online module freely to re-explore information already explained in the face-to-face sessions.

#### Data handling, storage and archiving

Data of participants will be handled confidentially and coded. The research data will be pseudo-anonymised by giving each participant a unique code. The code list, with both codes and names of the participants will only be accessible for the members of the research team and will be stored in a secured file. The handling of personal data will be done according to the General Data Protection Regulation. Data will be collected and stored in the cloud-based platform Castor EDC, and Labguru. Data and biological samples will be kept for 15 years to be able to perform additional analyses in case of new developments and insights. Participants can give their permission for this via the informed consent form.

### Statistical analysis

Data collected via questionnaires will be analysed using the software IBM SPSS V.29 for Microsoft Windows, data regarding the biological samples will be analysed using R software. The threshold for statistical significance is defined as <0.05.

For the primary outcome, pain NRS scores, repeated measures ANOVA will be conducted with time (T0 vs T2) as the within-participants variable, group (SC, SC + AIDI, SC + CBT, and SC + AIDI + CBT) as between-participant variable, and the pain NRS score as the dependent variable. If there are missing data, or a temporary study halt, a linear mixed model will be used instead of repeated measures ANOVA. Model fit will be assessed with residuals vs predicted plots and observed vs predicted plots. An intention-to-treat analysis will be followed in the case of follow-up losses. In this analysis, participants are analysed according to their original group assignment, regardless of whether they completed the treatment or dropped out, to minimise the risk of bias due to selective dropout. Given the random assignment of the intervention groups, a regression analysis on the pre-post change, adjusting for the pre-intervention score will be used to measure the main effect of the AIDI and CBT, and estimate interaction effects. Mediation analysis will be conducted to understand if and how secondary outcome measures mediate the effect of the intervention on the primary outcome.

To investigate the differences between women with and without endometriosis at baseline, a Welch’s ANOVA will be performed. This method is chosen to account for unequal group sizes and variances. In case the data are not normally distributed, a non-parametric Kruskal-Wallis test will be used.

### Data monitoring

Monitoring will take place following the Dutch NFU Richtlijn Kwaliteitsborging mensgebonden onderzoek.^39^ Because of the negligible risk of the study, only minimal monitoring will be performed by the Radboud Technology Centre for Clinical Studies. An initiation visit will be done before inclusion of the first patient, one monitoring visit will be planned and a close out visit. The monitoring will include completeness of the Trial Master File, availability of signed informed consent forms, verification of the in- and exclusion criteria, check of accuracy on the described SAE’s and a check on the data management in Castor EDC.

### Premature determination of the study

The study can be terminated prematurely if there is evidence of an unacceptable risk for trial subjects, if there is reason to conclude that it is not feasible to collect the data necessary to reach the study objectives and it is therefore not ethical to continue and in case of failure of the investigator and/or staff to follow either good clinical practice standards or to adhere to protocol requirements. The decision to end the trial prematurely will be made by the coordinating investigators in close collaboration with the principal investigator.

### Ethics and dissemination

The study protocol has been approved by the METC Oost-Nederland from Radboud University Medical Centre on 11 July 2024, and was registered on ClinicalTrails.gov on 13 March 2024 (registration number: NCT06332560). After the first initial approval by the METC Oost-Nederland, amendments were submitted, all of which were approved. The latest version of the protocol is version 7 (1 October 2025). This study will be conducted according to the principles of the Declaration of Helsinki (V.2013) and in accordance with the Medical Research Involving Human Subjects Act (WMO).

The sponsor/investigator has a liability insurance which is in accordance with article 7 of the WMO. Due to the nature of the interventions the METC did not require the sponsor to take out additional insurance to cover non-negligent harm associated with the study protocol. Prior to participation, participants are required to provide informed consent. The first participant was included on 4 October 2024, and the aim is to attain all inclusions by the end of 2026. If participants wish to respectively start or continue the AIDI/CBT after the completion of the study, they are instructed to contact their gynaecologist or general practitioner for a referral to a dietitian or psychological therapist.

The results will be widely disseminated through scientific peer-reviewed journals, and presented to a broad audience in scientific meetings, congresses, patient meetings, as well as in policy relevant forums.

## Supplementary material

10.1136/bmjopen-2026-116964 online supplemental file 1

10.1136/bmjopen-2026-116964 online supplemental table 1

## Data Availability

Data sharing not applicable as no datasets generated and/or analysed for this study.
