# Supplementary material for: Effectiveness of an anti-inflammatory diet intervention and cognitive behavioural therapy in endometriosis: protocol for a randomised controlled clinical trial
Source: BMJ Open. 2026 May 26;16(5):e116964. doi: 10.1136/bmjopen-2026-116964 (PMC13218105; doi:10.1136/bmjopen-2026-116964)
Supplement: online supplemental file 1 [file bmjopen-16-5-s001.docx]

**Supplementary Material 1: Informed Consent form PEARL study**

This form is related to the PEARL study: Pain in Endometriosis And the Relation to Lifestyle.

- I have read the participant information letter. I was able to ask questions, and my questions were answered to satisfaction. I had enough time to decide whether to participate in this study.
- I understand that participation is voluntary. I also do understand that I can decide at any time to withdraw from the study, or stop participating. I do not have to explain why I want to stop.
- I give the researcher permission to inform my general practitioner and the specialist treating me that I am participating in this study.
- I give the researcher permission to share any relevant unexpected findings from the study with my general practitioner or specialist that may be important for my health.
- I give the researchers permission to collect and use my data and biological material. The researchers will use this only to answer the research question of this study.
- I am aware that, for the purpose of monitoring the study, certain individuals may access all my data. These individuals are listed in the participant information letter. I give these individuals permission to access my data for this purpose.
- I understand that I must not become pregnant during the study.
- Please indicate with a yes or no in the table below.

| I give permission to store my data for use in other research, as stated in the information letter. | Yes ☐ | No ☐ |
| --- | --- | --- |
| I give permission to store my (remaining) biological material for use in other research, as stated in the information letter. The biological material will be stored for an additional 15 years. | Yes ☐ | No ☐ |
| I give permission to be contacted after this study to ask if I would like to participate in a follow-up study. | Yes ☐ | No ☐ |

- I want to participate in this study.

Name: ____________________________

Signature: ……………………… Date : __ / __ / __

-----------------------------------------------------------------------------------------------------------------

I declare that I have fully informed this participant about the mentioned study.

If any information becomes known during the study that could affect the participant’s consent, I will inform the participant in a timely manner.

Name researcher (or their representative):……………………………….

Signature:……………………… Date: __ / __ / __

-----------------------------------------------------------------------------------------------------------------

The participant will receive a complete information letter, along with a signed version of the consent form.
