# Supplementary material for: Effectiveness of an anti-inflammatory diet intervention and cognitive behavioural therapy in endometriosis: protocol for a randomised controlled clinical trial
Source: BMJ Open. 2026 May 26;16(5):e116964. doi: 10.1136/bmjopen-2026-116964 (PMC13218105; doi:10.1136/bmjopen-2026-116964)
Supplement: online supplemental table 1 [file bmjopen-16-5-s002.docx]

**Supplementary Table 1**. Anti-inflammatory diet intervention (AIDI) guidelines

| **Principle of the AIDI** | A predominantly plant-based diet with three to six regular meals a day. The AIDI is rich in the consumption of vegetables, fruit, legumes, nuts, whole grains, fish and adequate dairy intake. And it limits the intake of red and processed meat, alcohol, sugary drinks, salt, added sugars, and saturated fats.  Overall, subjects should have sufficient intake of macronutrients, vitamins and minerals. Salt (NaCl) intake should not exceed six grams per day. | | |
| --- | --- | --- | --- |
|  | | | |
| **Guidelines per food group** | | **Prefer** | **Avoid** |
| **Vegetables** | Minimum intake of 250 grams (six serving spoons) each day; eat at least twice a day. | Unprocessed fresh or frozen vegetables. | Canned or jarred vegetables containing added sugars and/or salt. |
| **Fruit** | 200 – 300 grams (2 to 3 pieces) of fruit every day, of which one serving (100 grams) of red fruit.  Maximum of 20 grams of dried fruits: dates, raisins. | Unprocessed fresh or frozen fruits.  Examples of red fruits: strawberries, blueberries, raspberries. | Canned fruit in syrup. |
| **Legumes** | Minimum intake of two servings a week. A serving is equal to 50 – 75 grams uncooked dried legumes. | Dried legumes, such as: lentils, chickpeas, beans (must be washed before cooking).  Canned or jarred legumes without added sugars and salt (rinsed with water). | Canned or jarred legumes with added sugars and/or salt, for example: white beans in tomato sauce. |
| **Nuts and seeds** | 25 grams (a handful) | Unsalted, unroasted nuts and/or seeds.  100% peanut butter or nut butter without any added sugars and salt. | Salted, roasted or sugared. |
| **Grains, potatoes and bread** | At least 90 grams a day | Wholegrain variants (e.g. wholegrain pasta, brown rice, quinoa, bulgur, oatmeal), unprocessed white or sweet potatoes, whole-wheat bread, muesli without added sugars. | White pasta and rice, processed potato products: fries, rösti e.g., white bread, buns and crunchy muesli with added sugars. |
| **Dairy** | Maximum of two to three portions (150-200 ml or grams) per day | Unsweetened dairy products like yoghurt, quark, Skyr, kefir and buttermilk (preferably semi-skimmed or skimmed).  Unsweetened dairy substitutes, preferable soy or pea based with added Calcium and vitamin B12. | Sweetened dairy products or dairy substitutes with added sugars and without added Calcium and vitamin B12. |
| **Cheese** | Maximum 30 grams a day. | Preferably 30+ cheese.  Hard cheeses like Grana Padano, Pecorino Romano, Manchego. | High fat cheeses like Brie, Camembert. |
| **Eggs** | Maximum of ten each week. |  |  |
| **Red meat** | Maximum of 100 grams (uncooked weight) per week. | Unprocessed, lean meat like beef or lamb. | Processed, red meat containing high amounts of saturated fats. |
| **Poultry** | Maximum of 200 grams (uncooked weight) per week. | Unprocessed, that has not been modified by curing, smoking or adding preservatives. | Processed poultry like sliced chicken breast for sandwiches, seasoned poultry. |
| **Fish** | At least one portion of 100 – 125 grams. | Fatty fish like salmon, mackerel, sardines, herring, trout. | Processed fish like canned fish in sour or sauce, fried fish, fish sticks, seasoned or breaded fish.  Smoked fish like smoked salmon is maximised to 100 grams every two weeks. |
| **Oils and fats** | Two tablespoons (10 – 20 grams) | (Extra virgin) vegetable oils, liquid cooking fats and soft margarines. | Butter, hard margarines, coconut oil. |
| **Liquids** | At least 1,5 to 2 liters a day. | Water, unsweetened tea, or unsweetened coffee. | Sugary beverages, alcoholic drinks. |

**Supplementary Table 2**. Cognitive behavioural therapy (CBT) protocol

| **Principle of the CBT** | | This CBT protocol can be applied to patients who experience pain symptoms as a result of endometriosis. The current CBT protocol can be used as a framework for the CBT. The psychologist is free to tailor the therapy specifically to the patients’ concerns and problems.  All CBT sessions will have a fixed layout: each session begins with a brief introduction of the session. Next, the homework assignments from the previous session are discussed (except in the first session). Then the themes of the particular session are explained. The patient and psychologist will together execute assignments to support positive coping skills. Finally, the participant and psychologist will decide on one or more homework assignments that should be carried out in preparation for the next session before concluding the session by a brief evaluation. | |
| --- | --- | --- | --- |
|  | | | |
| **Sessions** | | | **Explanation of session** |
| **1** | Consequences model and management of expectations towards the CBT. | | - First acquaintance between patient and therapist. Completing the consequence model: investigate the experienced consequences of pain caused by endometriosis on physical, behavioural, emotional, and social domain Identifying expectations of the AIDI and CBT interventions. - Introduction to pain education on acute and chronic pain. |
| **2** | Goal setting. | | - Setting of individual goals for CBT and adherence to diet based on the consequence model. - Continue on pain education. |
| **3** | Pain and behaviour. | | - Explanation of the interaction between behaviour and pain. - Investigate how the patient’s behaviour can have an impact on pain on the short and long term. - Introduction to Thought Record. - Investigate the patient’s ability to handle and cope with pain and stress. Examine the balance of burden and resilience. - Relaxation strategies. |
| **4** | Pain, thoughts and emotions. | | - Psycho-education on emotions and how they affect cognitions. - Investigate the patients’ cognitions in relation to experiencing pain symptoms. - Examine helpful cognitions. |
| **5** | G-scheme and hypervigilance. | | - Continue on the use of Thought Records. - Psycho-education on hypervigilance and catastrophizing. |
| **6** | Intimacy and sexuality. | | - The role of pain on intimacy and sexuality. |
| **7** | Final session. | | - Evaluation of CBT. - Repetition of topic of choice from the patient. - Discuss relapse prevention plan. |
