# Supplementary material for: Effectiveness of an anti-inflammatory diet intervention and cognitive behavioural therapy in endometriosis: protocol for a randomised controlled clinical trial
Source: BMJ Open. 2026 May 26;16(5):e116964. doi: 10.1136/bmjopen-2026-116964 (PMC13218105; doi:10.1136/bmjopen-2026-116964)
Supplement: Supplementary file 3 [file bmjopen-2026-116964.reviewer_comments.pdf]

PEER REVIEW HISTORY

BMJ Open publishes all reviews undertaken for accepted manuscripts. Reviewers are asked to complete a checklist review form and are provided with free text boxes to elaborate on their assessment. These free text comments are reproduced below.

ARTICLE DETAILS

Title (Provisional)

Effectiveness of an anti-inflammatory diet intervention and cognitive behavioural therapy in endometriosis: protocol for a randomised controlled clinical trial

Authors

Huijs, Emma; van der Sman, Loutje; Wirken, Lieke; Delcliseur, Heidi Sabine Mathilde Jeanne; Hameleers, Esther Gerdien; de Roos, Nicole; Van der Molen, Renate; Oosterman, Joukje M.; Viganò, Paola; Horne, Andrew; Working Group, EUmetriosis; Dolmans, M. M.; Hoogstad-van Evert, Janneke S.; Nap, Annemiek W.

VERSION 1 - REVIEW

**Reviewer**	**1**
**Name**	**Seyer-Hansen, Mikkel**
**Affiliation**	**Aarhus University, Department of Clinical Medicine**
**Date**	**22-Mar-2026**
**COI**	**None**

This is a very well written protocol for a large randomized trial on a much needed aspect of endometriosis treatment - the empowerment of the patients and the influence of lifestyle interventions. The knowledge is sparse today regarding both dietary recommendations and cognitive therapy. The idea of combining both in the same study makes good sense because it reflects the way many women with endometriosis seek supplementary treatment options. The accept from the study population should be high is suppose.

Only concern is is the 13-week study period is long enough to produce significant results on the primary outcome - level of pain.

VERSION 1 - AUTHOR RESPONSE

<u>Reviewer #1</u>

This is a very well written protocol for a large randomized trial on a much needed aspect of endometriosis treatment - the empowerment of the patients and the influence of lifestyle

interventions. The knowledge is sparse today regarding both dietary recommendations and cognitive therapy. The idea of combining both in the same study makes good sense because it reflects the way many women with endometriosis seek supplementary treatment options. The accept from the study population should be high is suppose.

Only concern is the 13-week study period is long enough to produce significant results on the primary outcome - level of pain.

Thank you for your compliments.

The study period is based on our previous feasibility study in which we investigated the effect of an anti-inflammatory diet in endometriosis. In this study we observed a significant change in pain scores, therefore we do believe that the study period of 13 weeks will be long enough to observe changes in the primary outcome.

If you have selected 'Yes' above, please provide details of any competing interests.: Not applicable.

The competing interests statement is mentioned in line 586-594.
